# Hôtel Dieu

**Published:** 1832-06

**Authors:** 


### HÔTEL DIEU.

*Induration of the Medullary Substance of the Brain, Disorganization of the Left Optic Thalamus, Hemiplegia of the Right Side, Contraction of the Right Arm, and total Loss of Intellect.**

On the 7th of August, a man, still vigorous and in good condition, about sixty-six years of age, was placed under the care of M. Guéneau de Mussy, having been ill, as was reported, four or five days. He did not speak, had his eyes constantly closed, and the eyelids squeezed together; mouth slightly open, but the tongue not put out, even when it was attempted to be seen. The right arm was rigidly contracted, and the same side of the body motion-

* Lancette Français.

less: sensibility remained, but it was dull; when the arm or the leg of this side were pinched, he moved them away. The pulse hard, full, and slow; respiration free; bowels confined. He was bled twice within twenty-four hours; had leeches applied behind his ears; sinapisms to his feet, and blisters to the calves of his legs; purgative clysters were likewise administered, and the patient kept on broth diet.

This treatment, though active, was not followed by any beneficial effects, and it was only then learned that the symptoms had existed for three weeks. Active measures were no longer persisted in, as the case was deemed desperate. Immediately food was given to him, he devoured it with an avidity which shewed that it would be wrong to add to his misery, by allowing him to suffer from the pangs of hunger. Still he never asked for victuals: the only words he uttered were imprecations on the attendants who cleaned him. He was habitually costive, but from time to time he had occasional copious evacuations.

During the thirty-nine days he remained in the hospital, he shewed the traits of ungovernable temper. Many times he bit the nurses who changed his linen, and endeavoured with his left hand, which remained healthy, to seize any one who approached him, and struck at them with great violence. During the first few days after his admission, spasms were observed in the paralytic limb.

The first symptoms of sinking that we noticed were diminution of the contractions of the left side of the thorax, more profound and constant heaviness, and loss of appetite; pulse scarcely perceptible during the last eight and forty hours. On the morning of the day he died, his respiration became anxious, and the embarrassment having increased all day, towards evening he expired in a state of asphyxia, occasioned by the accumulation of bronchial mucus.

The post-mortem examination discovered a considerable serous effusion at the base of the brain, and also in the ventricles. The medullary substance was remarkable for its density, which was so great that it contracted when let go after it had been stretched; and the brain, when removed from the cranium, and placed upon a plane surface, perfectly retained its figure. The white fibres of the corpus striatum might be separated from the grey matter. The floor of the lateral ventricles being laid bare, it was easy to perceive that the left optic thalamus presented a very considerable alteration: the medullary layer, on its upper surface, was thinner than natural, perforated at many points, and of a dirty yellowish hue. These openings led to a cavity formed in the substance of the optic thalamus, half filled with thick yellowish serum, and with a deposit of a yellow matter almost becoming brown. This colour did not extend into the thickness of the substance of the brain, which remained healthy. The morbid alteration was strictly confined to the optic thalamus, and yet the opposite side of the body was paralysed, and the arm rigidly bent.

